# Risk Assessment of Severe Congenital Anomalies of the Kidney and Urinary Tract (CAKUT): A Birth Cohort

**DOI:** 10.3389/fped.2019.00182

**Published:** 2019-05-14

**Authors:** Chryso P. Katsoufis, Marissa J. DeFreitas, Juan C. Infante, Miguel Castellan, Teresa Cano, Daniela Safina Vaccaro, Wacharee Seeherunvong, Jayanthi J. Chandar, Carolyn L. Abitbol

**Affiliations:** ^1^Division of Pediatric Nephrology, Department of Pediatrics, Miller School of Medicine, University of Miami, Miami, FL, United States; ^2^Holtz Children's Hospital, Jackson Health System, Miami, FL, United States; ^3^Miami Transplant Institute, Jackson Health System, Miami, FL, United States; ^4^Department of Radiology, Miller School of Medicine, University of Miami, Miami, FL, United States; ^5^Pediatric Urology, Nicklaus Children's Health System, Miami, FL, United States; ^6^Department of Pediatrics, Miller School of Medicine, University of Miami, Miami, FL, United States

**Keywords:** neonatal CAKUT, nadir creatinine, peak creatinine, cystatin C, biomarkers of early CKD

## Abstract

Recent advances in the early diagnosis of fetal CAKUT with an increase in fetal surgical interventions have led to a growing number of neonatal survivors born with severe renal dysfunction. This, in turn, has required the development of multi-disciplinary treatment paradigms in the individualized management of these infants with advanced stage kidney disease from birth. Early multi-modal management includes neonatal surgical interventions directed toward establishing adequate urine flow, respiratory support with the assessment of pulmonary hypoplasia, and establishing metabolic control to avoid the need for dialysis intervention. The development of specialized imaging to assess for residual renal mass with non-invasive 3-dimensional techniques are rapidly evolving. The use of non-radioactive imaging offers improved safety and allows for early prognostic-based planning including anticipatory guidance for progression to end stage renal disease (ESRD). The trajectory of kidney function during the neonatal period as determined by peak and nadir serum creatinine (SCr) and cystatin C (CysC) during the first months of life provides a guide toward individualized prospective management. This is a single center experience based on a birth cohort of 42 subjects followed prospectively from birth for an average of 6.1 ± 2.8 years at the University of Miami/Holtz Children's Hospital during the past decade. There was an 8:1 male: female ratio. The birth cohort was divided into 3 subgroups according to CKD Stages at the current age: CKD 1–2 (Group 1) (eGFR ≥ 60 ml/min/1.73 m^2^) (*N* = 15), CKD stage 3–5 (Group 2) (eGFR ≤ 59 ml/min/1.73 m^2^) (*N* = 12), and ESRD—Dialysis and/or Transplantation (Group 3) (*N* = 15). A neonatal CysC >3.0 mg/L predicted progression to ESRD while a nadir SCr >0.6 mg/dL predicted progression to CKD 3–5 with the highest specificity and sensitivity by ROC-AUC analysis (*P* < 0.0001). Medical management was directed toward nutritional support with novel formula designs, early introduction of growth hormone and strict control of mineral bone disorder. One of the central aspects of the management was to avoid dialysis for as long as feasible with a primary goal toward pre-emptive transplantation.

## Introduction

Congenital anomalies of the kidney and urinary tract (CAKUT) are the most common cause of end stage renal disease (ESRD) in children accounting for 50–70% of those who begin renal replacement therapy (RRT) worldwide (1–4). The great majority are diagnosed prenatally or within the first months of life. Most challenging is the early management of these infants with bilateral and severe CAKUT who have significant renal compromise at birth (3–5). The natural history of severe neonatal CAKUT remains incompletely understood with respect to the demographic and biological “predictors” of progression to chronic kidney disease (CKD). Moreover, consistent with the developmental and physiologic renal accommodations to the extra-uterine environment, there is a natural improvement in glomerular filtration rate (GFR) from birth throughout the first 2 years of life (4–8). The management of infants with severe CAKUT must consider and optimize this renal development in hopes of preserving nephron reserve to extend the longevity of individual renal function.

The aim of this study was to assess the natural progression and neonatal characteristics of a unique birth cohort of infants with severe CAKUT who were followed prospectively at our center at the University of Miami/ Holtz Children's Hospital during the past decade.

A multidisciplinary team focused on early diagnosis, conservative surgical intervention, analysis of “predictive” markers of progression, and preservation of renal function and growth during the first years of life with a goal toward pre-emptive transplantation when feasible. Although a retrospective review, this is an initial report of a birth cohort with the intent of continued prospective observational follow-up through the first 2 decades of life. The study was approved by the University of Miami Institutional Review Board, and patient and family privacy were assured under conditions of the Health Insurance Portability and Accountability Act.

## Patients and Methods

This is a retrospective cross-sectional analysis of infants managed at our center from January 1, 2004 through December 31, 2018 who were identified with severe CAKUT either prenatally or within the first 3 months of life. Criteria for inclusion were a diagnosis of “severe” CAKUT (8, 9) with either a unilateral or bilateral renal phenotype that resulted in fetal oligohydramnios, lower urinary tract obstruction (LUTO) and/or renal hypo-dysplasia resulting in neonatal renal dysfunction. Only infants who had neonatal data including birth history, birth weight, gestational age and neonatal renal functional markers were included in the birth cohort. Infants who died within the first week of life were not considered in this cohort.

### Neonatal Markers of Renal Function

Neonatal markers of renal function that were used as prognostic indicators of post-natal progression of CKD included peak and nadir serum creatinine (SCr) and cystatin C (CysC). SCr was measured by the enzymatic method and was patterned from the initial post-natal assay drawn during the first week of life but after the first 48 h and sequentially until the time of discharge from the hospital. Peak SCr was the maximum rise in SCr after birth and the nadir SCr was the lowest level recorded during the first 3 months of life. Serum CysC levels were assessed by the central laboratory at the same time as the SCr with a particle-enhanced immunonephelometric immunoassay (Dade-Behring, Deerfield, Illinois). The neonatal CysC levels were assayed during the first 3 months of life, usually prior to discharge from the neonatal hospitalization and concurrent with the “nadir” SCr. Subsequent assays of both SCr and CysC were obtained sequentially to follow concurrent changes in the biomarkers and to utilize these for assessment of progression of CKD. As this was a retrospective analysis, all assays were at the discretion of the treating neonatologist or nephrologist and did not follow a strict protocol or timeline. However, the average time in days for the assays was determined and compared among the groups.

Renal function was defined by estimating glomerular filtration rate (eGFR) according to contemporary estimating equations (10). The neonatal eGFR was calculated from the CysC as recommended in infants and subjects with low muscle mass (10, 11) as follows:

$$\begin{array}{l} {\text{eGFR}}_{\text{cys}}={70.69\;(\text{CysC})}^{-0.931} \end{array}$$

Subsequent assessment of renal function used both the single and combined eGFR equations from the revised Schwartz/CKiD report (10). This was the bedside Schwartz equation and the average of the SCr and CysC equations without the urea nitrogen or gender corrections as follows:

$$\begin{array}{l} {\text{eGFR}}_{\text{cr}}=41.3\;(\text{height}(\text{meters}))/\text{SCr}(\text{mg}/\text{dl}) \end{array}$$

All eGFR equations are given in milliliters/min/1.73 m^2^ (ml/min/1.73 m^2^).

### Classification of Renal Function by CKD Stage

Patients were categorized into 3 subgroups according to their stage of CKD at their age at the time of reporting. These were Group 1, early stage CKD (eGFR ≥60 ml/min/1.73 m^2^), Group 2, intermediate and pre-dialysis stage CKD (eGFR ≤59 ml/min/1.73 m^2^) and Group 3, ESRD (Dialysis and/or Transplantation). At the time of reporting, those on dialysis or those who were post-transplant were categorized into the ESRD category regardless of the renal allograft function. This subgroup classification was chosen uniquely to this cohort because of their young age and the focus in management of maintaining young patients with advanced CKD off dialysis if their clinical condition allowed pre-emptive transplantation without prior dialysis.

### Management Paradigms

Infants with CAKUT are managed through a multidisciplinary treatment paradigm for individualized management of their advanced CKD from birth. This was coordinated through a multifaceted clinical team including expert pediatric nephrologists, nurse specialists, a renal pediatric dietician, pediatric surgeons/ urologists, and specialized imaging services. The complex management of the patients described in this report was based on published guidelines (12–14) and experiential application of evolving treatment technologies including those related to drug development as well as dialysis and surgical technologies (15–19). Care was focused on controlling metabolic bone disease, hyperparathyroidism, maintaining growth, fluid and electrolyte, acid-base balance, albuminemia and target hemoglobin at every stage of CKD (5, 12–14).

### Nutrition and Growth

Growth was monitored from birth with close recording of weight, length and head circumference by standard anthropometric techniques and tracked on standardized Center for Disease Control (CDC) growth charts (20) including *Fenton* adjustments for prematurity (21).

Nutritional management was based on published guidelines and our pediatric renal nutrition specialist (22, 23). Nutritional aims were to provide 100% of estimated nutrient requirements for energy and supplemental protein as indicated for dialysis losses. Gastrostomy (G-tube) feedings were implemented early in the course to maintain adequate weight gain and prior to the anticipated need for dialysis (22–24). A predominantly whey-protein based formula was used during the first years of life with individualized adaptations to maintain electrolyte and nutritional balance to support growth. Transitions were made to oral intake when the patient matured adequately to tolerate and consume adequate nutrients by mouth. Recombinant growth hormone (rhGH) was initiated as early as 9 months of age for indications of substandard growth velocity or loss of linear growth z-score (25).

### Mineral Bone Disorder and Hyperparathyroidism

The control of the mineral bone disorder with instances of secondary hyperparathyroidism (sHPT), sometimes refractory to conventional treatment regimens, required the development of unconventional treatment regimens including activated vitamin D analogs and calcimimetics (6, 7, 13–17, 26). Monitoring included frequent calcium, phosphorus and intact parathyroid hormone (iPTH) levels as well as bone imaging and clinical assessment of bone pain, fractures and deformities (13, 14).

### Urologic Intervention and Management

Coordination of urologic and medical management was paramount to the successful outcomes in these complex patients. Prenatal interventions occurred in a discrete number of the current birth cohort; but this is not a primary focus of the current report (27, 28). Importantly, early post-natal management in each subject included relief of obstruction and establishment of free urine flow. This was accomplished in each patient after clinical imaging and urologic intervention. Subsequent surgeries were predicated on the need to establish constant urine flow, avoid infection and establish long-term urinary and bladder drainage. Preparation for transplantation included bladder augmentation when indicated and establishment of adequate long-term urinary drainage for the renal allograft (27, 28).

### Dialysis and Transplantation

With a focus on patient longevity and our personal center experiences (4–8), we tried to avoid dialysis in our severe CAKUT patients if they had good daily urine diuresis and could be adequately maintained in metabolic balance with good growth. As per current international recommendations, peritoneal dialysis (PD) was the preferred initial dialysis treatment modality followed by hemodialysis when necessary (4–8).

### Co-morbid Conditions

Recognition of co-morbidities included pulmonary hypoplasia, infection, concurrent syndrome/genetic diagnoses, and malignancy. These were considered in the multiple regression analysis of co-morbidities on mortality and the progression of CKD.

### Specialized Imaging

Specialized imaging to assess for residual renal mass with non-invasive 3-dimensional techniques were developed. This included the use of non-radioactive imaging for improved safety and to allow for early prognostic-based planning. Vascular imaging with computed tomography (CT)-angiography and magnetic resonance (MR) urography and angiography allowed for pre-surgical planning, including urologic reconstruction and vascular anatomical planning for renal transplantation (28, 29). The use of specialized contrast agents such as or ferumoxytol (Feraheme®) for MR angiography to avoid gadolinium associated nephrogenic systemic fibrosis in ESRD patients should also be considered if resources are available (29, 30).

### Statistical Analyses

All data sets were tested for normality with the D'Agostino and Pearson omnibus normality test. Data were expressed as the mean ± standard deviation (SD) or median with interquartile range (IQR) as appropriate. Intergroup comparisons were tested with ANOVA. Post-test comparisons for significance were performed by the Kruskal-Wallis test for non-parametric data and by the Bonferroni method for parametric data as appropriate. Differences between 2 groups were analyzed by the Student *t*-test. Proportional differences were tested with the Fisher exact test with hazard ratios and 95% confidence intervals (95%CI). The paired *t*-test was used to compare growth prior to and after transplantation. Receiver operating characteristic (ROC) and area under the curve (AUC) analyses were used to analyze the sensitivity and specificity of the neonatal biomarkers to predict the early progression to advanced CKD (Groups 2 and 3) and ESRD (Group 3) alone. The threshold was taken as the value of the marker that had the highest sensitivity at a specificity >85% with a significant likelihood ratio. The patient and renal survival by age in years were analyzed by Kaplan-Meier survival plots comparing curves with log-rank and Wilcoxan-Gehan tests for trend with calculation of hazard ratios.

Univariate correlations were performed with Pearson correlation coefficient (r). Multivariate linear regression analysis was used to determine the relative impact of mineral bone disease, co-morbid diagnoses, neonatal peak and nadir SCr, surgeries and infections on mortality and progression of CKD. GraphPad Prism (GraphPad Software, Inc, La Jolla, California) was used to perform the statistical analyses and to construct the graphs. A *p* < 0.05 was considered significant.

## Results

From a total of 52 subjects born during the study period with a diagnosis of severe CAKUT, 42 met inclusion criteria and had adequate neonatal data to predict progression of CKD over a median of 5.5 years (range 7 months to 11.8 years). The 10 exclusions were for inadequate birth data and lack of follow-up (*N* = 9) or lack of advanced neonatal renal dysfunction (*N* = 1). No infant who died during the first week of life was considered for inclusion. There was no infant born during this time period that was considered for dialysis during the first month of life. Table 1 provides the demographic characteristics according to primary diagnosis, race/ethnicity, gender, birth weight and gestational age, current age and CKD Stage. There was a male predominance with an 8:1 male:female ratio with 37 males (88%) and only 5 females. The racial/ethnic distribution was predominantly African American/Hispanic (81%) consistent with the demographic of the urban communities served by this university-affiliated public referral hospital in South Florida. Fetal surgical intervention was performed in 15 males (36%) with the predominant diagnosis of posterior urethral valves (PUV) (*N* = 24) and urethral atresia with Prune Belly syndrome (PBS) (*N* = 7). The primary diagnosis in the females was VACTERL association (*N* = 4) (vertebral, anal, cardiac, tracheo-esophageal fistula, renal, limb anomalies). Other diagnoses included bilateral hypodysplasia (*N* = 5), bilateral ureteropelvic junction obstruction (*N* = 1) and bilateral ureteroceles with bladder outlet obstruction (*N* = 1).The cohort was divided into 3 groups according to their eGFR at the time of reporting at an average age of 6.1 ± 2.8 years. They were evenly distributed into Group 1 (*N* = 15); Group 2 (*N* = 12) and Group 3 (*N* = 15).

**Table 1:** Patient demographics.

**Patient demographics**, ***N*** **=** **42**		
Age (years)	Mean ± SD	5.8 ± 2.9
Race, *N* (%)		
	Caucasian	8 (19%)
	Hispanic	9 (21%)
	African	25 (60%)
Gender, *N* (%)		
	Male	37 (88%)
	Female	5 (12%)
Birth weight [grams ± SD]		2,712 ± 660
Gestational Age [weeks ± SD]		36 ± 3
CKD Stage, *N* (%)		
	Group 1	15 (36%)
	Group 2	12 (28%)
	Group 3	15 (36%)
Primary Diagnosis, *N* (%)		
	Posterior Urethral Valves	24 (57%)
	Prune Belly Syndrome	7 (17%)
	Bilateral Hypodysplasia	5 (12%)
	VACTERL	4 (10%)
	Bilateral UPJ	1 (2%)
	Ureteroceles with BOO	1 (2%)

*SD, Standard deviation; CKD, Chronic kidney disease; ESRD, End Stage Renal Disease; VACTERL, vertebral defects, anal atresia, cardiac defects, tracheo-esophageal fistula, renal anomalies, and limb abnormalities; UPJ, ureteropelvic junction obstruction; BOO, Bladder outlet obstruction*.

### Neonatal Characteristics as Predictors of CKD Progression

Table 2 summarizes the neonatal characteristics by gender and current CKD stages. Current age was similar for all CKD stages. Gestational age (34 ± 2 vs. 37 ± 3 weeks; *p* < 0.01) and birth weight (2,463 ± 550 vs. 2,964 ± 680 grams; *p* < 0.01) were significantly less in those who had early progression to ESRD (Group 3) compared to those with lower stage CKD (Group 1), respectively. Those with intermediate stage CKD (Group 2) who did not require early dialysis or transplantation were intermediate between the other 2 groups in both birth weight and gestational age. Although there was a trend for the females, compared to the males, to be older [current median age: 8.0 (IQR 5, 10) vs. 5.6 ± 2.8 years] and to have lower birth weights [median 2,150 (IQR 2,108, 2,450) vs. 2,775 ± 676 grams] and lower GA [median 34 (IQR 32, 36) vs. 36 ± 3 weeks], the differences did not reach significance.

**Table 2:** Neonatal characteristics as predictors of progression to CKD.

	**Age (Years)**	**GA (Weeks)**	**Birth weight (grams)**	**Growth Current SDS**	**Peak neonatal SCr (mg/dL)**	**Nadir neonatal SCr (mg/dL)**	**Neonatal CysC (mg/L)**	**Neonatal eGFRcys ml/min/1.73 m^**2**^**
**NEONATAL CHARACTERISTICS AS PREDICTORS OF OUTCOME MEASURES**								
**CAKUT-by CKD Category (*****N*** **=** **42)**								
Group 1 (*N* = 15)	6.1 ± 2.8	37 ± 3^**^	2,964 ± 680^*^	0.4 ± 1.6^*^	1.3 ± 0.9^¶^^**^	0.4 ± 0.1^¶^^**^	1.5 ± 0.5^¶^^**^	48 ± 9^¶^^**^
Group 2 (*N* = 12)	6.4 ± 3.1	36 ± 5	2,653 ± 824	−0.5 ± 1.4	3.2 ± 1.3^**^	1.4 ± 0.9^**^	2.8 ± 1.0^**^	31 ± 13^**^
Group 3 (*N* = 15)	6.3 ± 2.7	34 ± 2^**^	2,463 ± 550^*^	−1.1 ± 1.4^*^	4.0 ± 1.1^¶^	2.1 ± 0.8^¶^	4.0 ± 0.7^¶^^**^	20 ± 3^¶^^**^
**Females vs. Males**								
Females (*N* = 5)	8.0 (5,10)	34 (32,36)	2,150 (2,108, 2,450)	−1.4 (−2.4, −0.9)	3.7 (1.7, 4.0)	1.9 (0.6, 2.2)	3.7 (1.9, 3.9)	21 (20, 46)
Males (*N* = 37)	5.6 ± 2.8	36 ± 3	2,775 ± 676	−0.3 ± 1.6	2.7 ± 1.7	1.2 ± 1.0	2.6 ± 1.3	34 ± 15

*CAKUT, Congenital anomalies of the kidney and urinary tract; CKD, chronic kidney disease; GA, gestational age; SCr, serum creatinine; CysC, cystatin C; Mean ± SD, Standard deviation; median (IQR), interquartile range; mg/dL, milligrams/deciliter; mg/L, milligrams/liter; ESRD, End Stage Kidney Disease (Dialysis and/or Transplantation); eGFRcys, estimated glomerular filtration rate by cystatin C equation; SDS, standard deviation score; Tx, transplant; PETx, pre-emptive transplant*.

* *p < 0.05 significantly different from the similarly marked variable*.

** *p < 0.01 significantly different from the similarly marked variable*.

¶ *p < 0.001 significantly different from similarly marked variable*.

Age at the time of assays of biomarkers was measured in days. The time to peak SCr was a median of 6 (IQR 2, 7) days with a nadir SCr at 42 ± 26 days. The neonatal cystatin C was assayed at an average of 36 ± 29 days. There was no significant difference between the groups in terms of days of the assays and all were well within the first 3 months of life.

Peak and nadir neonatal SCr were significantly lower in the early stage CKD (Group 1) (1.3 ± 0.9 and 0.4 ± 0.1 mg/dl, respectively) compared to those who had early progression to CKD (Group 2) (*p* < 0.01) and ESRD (Group 3) (*p* < 0.001). The peak and nadir SCr did not differentiate between the more advanced stages of CKD progression. In contrast, neonatal CysC was significantly different for each of the levels of CKD progression by ANOVA (*p* < 0.001). Mean values for neonatal CysC by progressive CKD stages were: Group 1: 1.5 ± 0.5 mg/L; Group 2: 2.8 ± 1.0 mg/L and Group 3: 4.0 ± 0.7 mg/L. Similarly, the neonatal eGFRcys neonatal CysC differentiated the progression to CKD with an eGFRcys in the neonatal period of 48 ± 9, 31 ± 13, and 20 ± 3 ml/min/1.73 m^2^ predicting CKD 1–2, 3–5, and ESRD, respectively, at the time of follow up.

**Figure 1:**
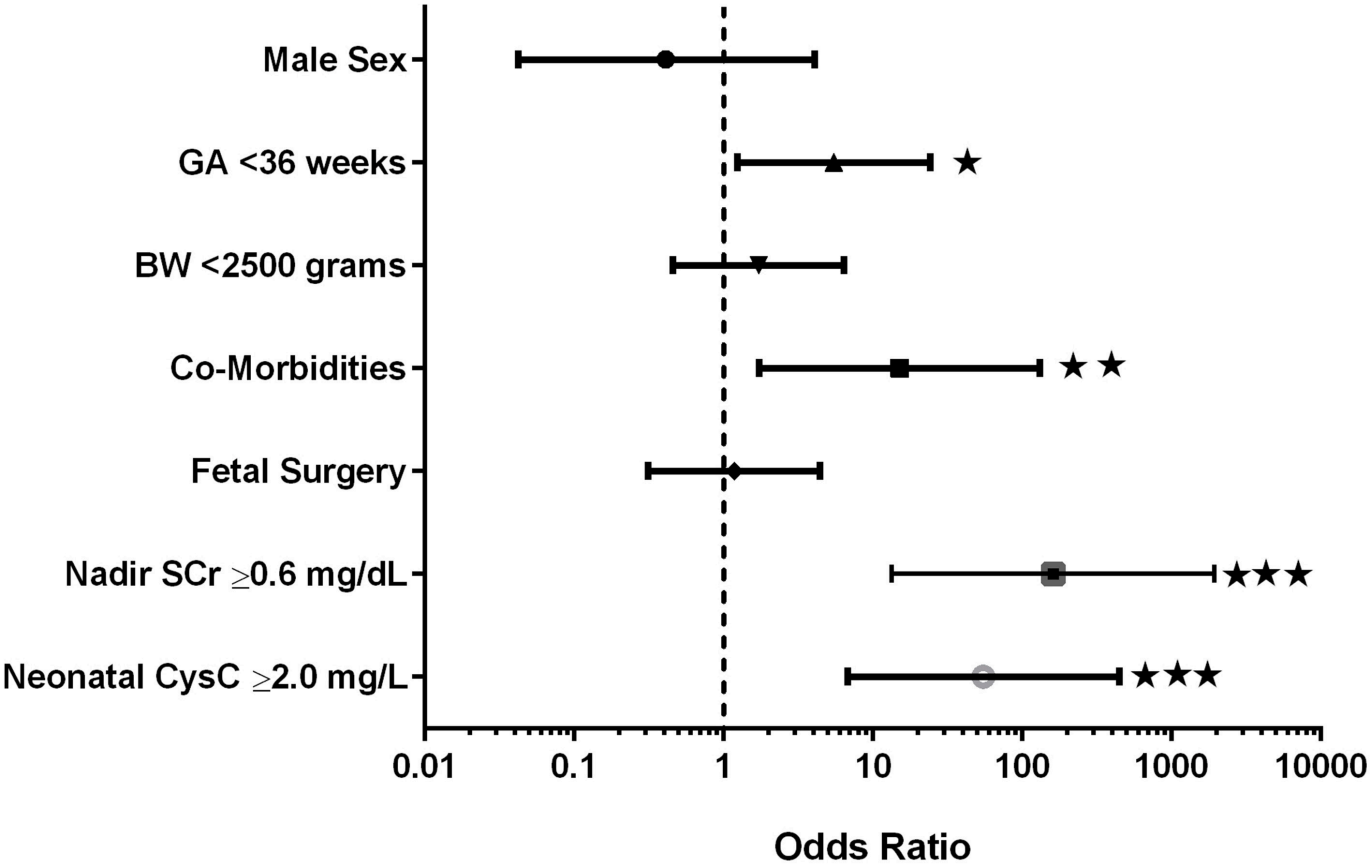
Odds ratios for prediction of early progression to advanced kidney disease. ⋆*p* < 0.05; ⋆⋆*p* < 0.01; ⋆⋆⋆*p* < 0.001.

Figure 1 provides the odds ratios for demographic characteristics affecting early progression to advanced CKD including gender, birth weight and GA, co-morbidities and fetal surgery as well as the lowest thresholds for neonatal nadir SCr and CysC. Importantly, male gender and fetal surgery were not significant predictors of early progression. Preterm birth with GA <36 weeks was a predictor of early progression; while low birth weight <2,500 grams was not. Co-morbidities which included lung hypoplasia and congenital anomalies involving other organ systems were a significant predictor of progression. The lowest thresholds for SCr (≥0.6 mg/dL) and Cystatin C (≥2.0 mg/L) were included in the analysis and proved to be the most significant predictors of progression to early CKD. Multiple regression analysis using these same co-variants was highly significant (*p* < 0.0001; *R*^2^ = 72.7%) with the main variants being nadir SCr >0.6 mg/dL [*p* < 0.001; β coefficient: 1.38 (95% CI: 0.9 to 1.9)] and growth (current sds) [*p* = 0.03; β coefficient: −0.13 (95% CI: −0.2 to −0.1)].

**Figure 2:**
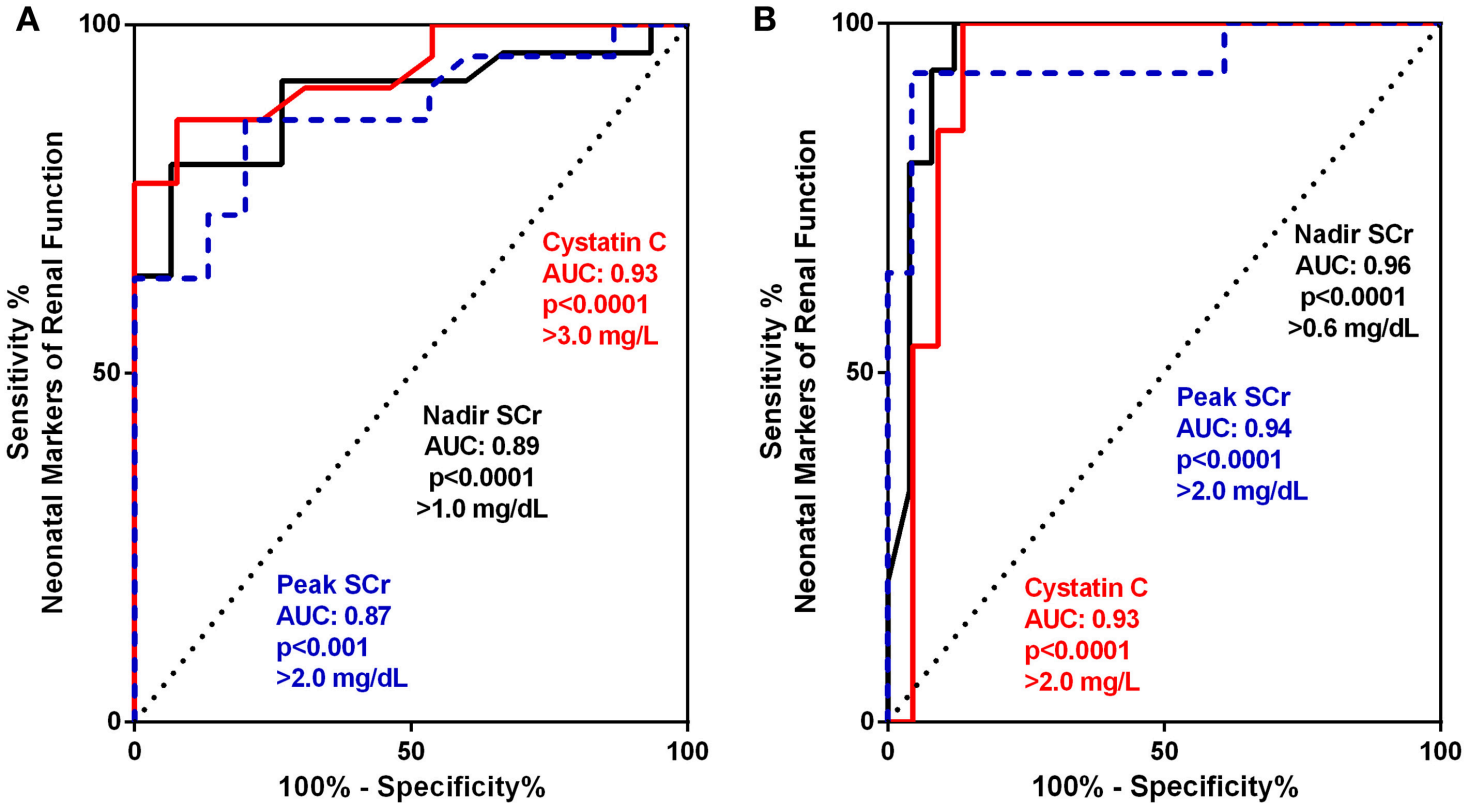
**(A,B)** Receiver operating curve (ROC) and area under the curve (AUC) for biomarker assessment of prediction to early progression to end stage kidney disease (ESRD) (Group 3) **(A)** and advanced chronic kidney disease (CKD 3–5 + ESRD) Group 2 and 3 **(B)**.

For a more granular assessment of the sensitivity and specificity of these neonatal biomarkers toward prediction of the progression of CKD in these severe CAKUT patients, ROC-AUC analyses were performed and are detailed in Tables 3A,B and Figures 2A,B. The ROC-AUC for prediction of ESRD alone is Table 3A, and Figure 2A shows that CysC had the greatest AUC = 0.93 with a sensitivity of 77% and a specificity of 92% (*p* < 0.0001). The defining threshold was a CysC ≥3.0 mg/L. Figure 2B shows the ROC-AUC analyses for early progression to CKD 3–5 and ESRD. In these analyses, the nadir SCr had the highest AUC = 0.96 with a threshold value of SCr ≥0.6 mg/dL (*p* < 0.0001). The range of nadir SCr was very narrow with a SCr ≥ 0.7 mg/dL having 100% sensitivity and 88% specificity. The minimum CysC threshold was ≥2.0 mg/L.

**Table 3:** Receiver operating curve (ROC) and area under the curve (AUC) analyses for biomarker assessment of prediction to early progression to end stage kidney disease (ESRD) (Group 3) **(A)** and advanced chronic kidney disease (CKD 3–5 + ESRD) (Groups 2 and 3) **(B)**.

**Parameter**	**AUC**	**Defining threshold**	**Sensitivity (%)**	**Specificity (%)**	**Likelihood ratio**	***P*-value**
**(A) Neonatal Predictors of Progression to ESRD**						
Peak SCr (mg/dL)	0.87	≥ 2.0	64	93	9.5	<0.001
Nadir SCr (mg/dL)	0.89	≥ 0.6	64	93	9.6	<0.0001
		≥1.0	80	93	12.0	
Cystatin C (mg/L)	0.93	≥ 3.0	77	92	10.1	<0.0001
**(B) Neonatal Predictors of Progression to CKD 3-5** **+** **ESRD**						
Peak SCr (mg/dL)	0.94	≥ 2.0	93	96	21.4	<0.0001
Nadir SCr (mg/dL)	0.96	≥ 0.6	93	92	11.7	<0.0001
		≥ 0.7	100	88	7.8	
Cystatin C (mg/L)	0.93	≥ 2.0	85	91	9.3	<0.0001

*ESRD, End Stage Renal Disease; SCr, serum creatinine; AUC, area under the curve*.

### Patient and Renal Survival by CKD Group

**Figure 3:**
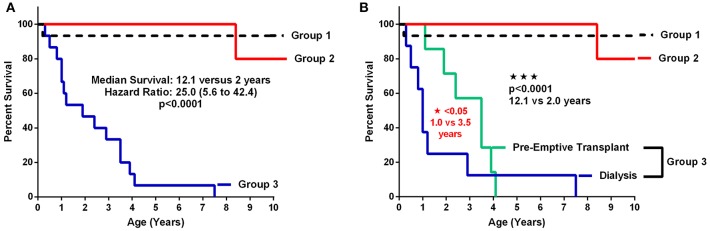
**(A,B)** Patient and renal survival curves according to Stage of CKD. **(A)** shows the survival curves of the 3 major subgroups comparing early stage and pre-dialysis CKD (Groups 1 and 2) vs. ESRD (Group 3). **(B)** shows the survival curves with the advanced CKD (Group 3) divided into the dialysis patients vs. those who were managed conservatively without dialysis until pre-emptive transplantation (TX: transplantation).

Patient and renal survival were plotted by Kaplan-Meier survival curves according to the 3 groups of patients defined by their level of CKD at the age of initial retrospective assessment. The 3 groups were CKD Stage 1–2 (Group 1); CKD Stage 3–5 (Group 2) and CKD Stage 5D + Transplant (ESRD) (Group 3). The ESRD was subdivided into those who went to early dialysis and those who were managed conservatively to pre-emptive transplant. Figure 3A shows the survival for the 3 groups with a significant (*p* < 0.0001) difference between CKD Stages 1–5 compared to the ESRD group with a median renal survival of 12.1 years for early stage CKD compared to 2 years for the advanced stages of CKD. When the ESRD group was divided into those who required early dialysis vs. those who were managed off dialysis until pre-emptive transplantation, the survival curves were also significantly (*p* < 0.05) different (Figure 3B). For those who were managed off dialysis, the time to pre-emptive transplantation was a median of 3.5 years compared to 1 year for those who required early dialysis.

### Growth and Nutrition

Growth was monitored at each stage of CKD and in accordance with control of the sHPT. The main determinants of growth by multiple regression analysis were birth weight and CKD stage (*R*^2^ = 33.4%; *p* = 0.02). Almost half (13/27 = 48%) of those with advanced CKD (Groups 2 and 3) and 73% (11/15) with ESRD (Group 3) had placement of a G-tube to maintain adequate nutrition. Growth hormone was prescribed for 19 of the cohort (45%) and in 87% (13/15) with advanced stage ESRD.

**Figure 4:**
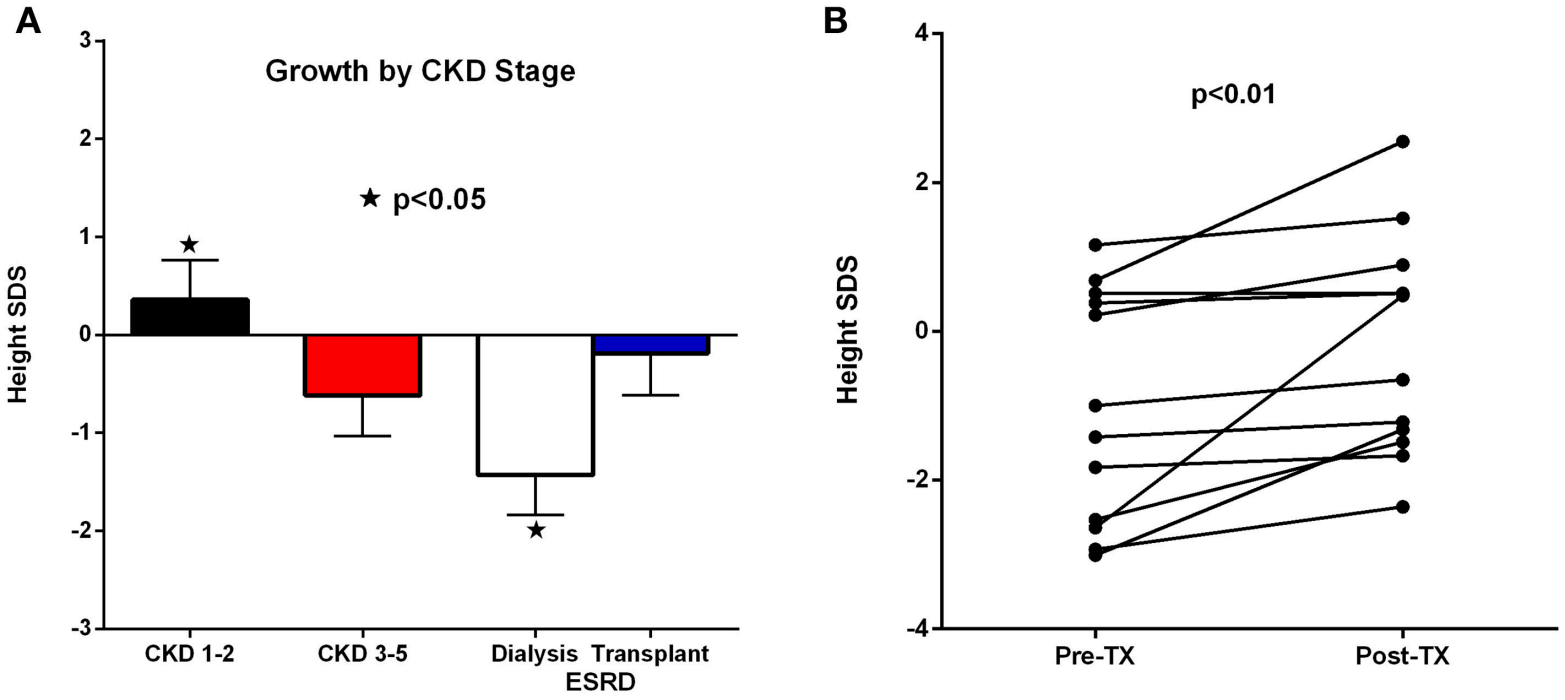
**(A,B)** Linear growth by chronic kidney disease (CKD) stage **(A)** and growth pre and post-transplant **(B)**.

Figure 4A demonstrates the growth of the subjects according to CKD stage. The ESRD stage is divided into those on dialysis vs. those after pre-emptive transplantation. The poorest growth was in those patients who required dialysis; whereas, those who underwent transplantation grew at similar rates to those with CKD 1–2 (Group 1). Figure 4B shows the effect on growth after transplantation with significant improvement (*P* < 0.01).

### Mortality and Co-morbid Conditions

Two subjects died at 1 and 21 months of age, respectively. The 1 month old infant had PUV and was status post valve ablation. He died of urosepsis with normal renal function. The other demise was a 21 month old with PUV who was on peritoneal dialysis from 9 months of age. He died of fungal peritonitis complicated by multiorgan failure with hepatic toxicity and respiratory failure. Both of these losses were attributed primarily to infectious complications of the primary disease.

### Co-morbid Conditions

Co-morbidities were divided into genetic/syndromic and/or fetal/developmental abnormalities such as pulmonary hypoplasia and pulmonary hypertension related to oligohydramnios, infectious complications related to urinary tract obstruction, and hormonal/metabolic complications related to renal dysfunction. Seventeen of the 42 subjects had co-morbid conditions (40.5%). As shown in Figure 1, the presence of any co-morbidity contributed significantly to the early progression of CKD (HR: 15.1;95% CI: 1.7–131; *p* = 0.006).

**Figure 5:**
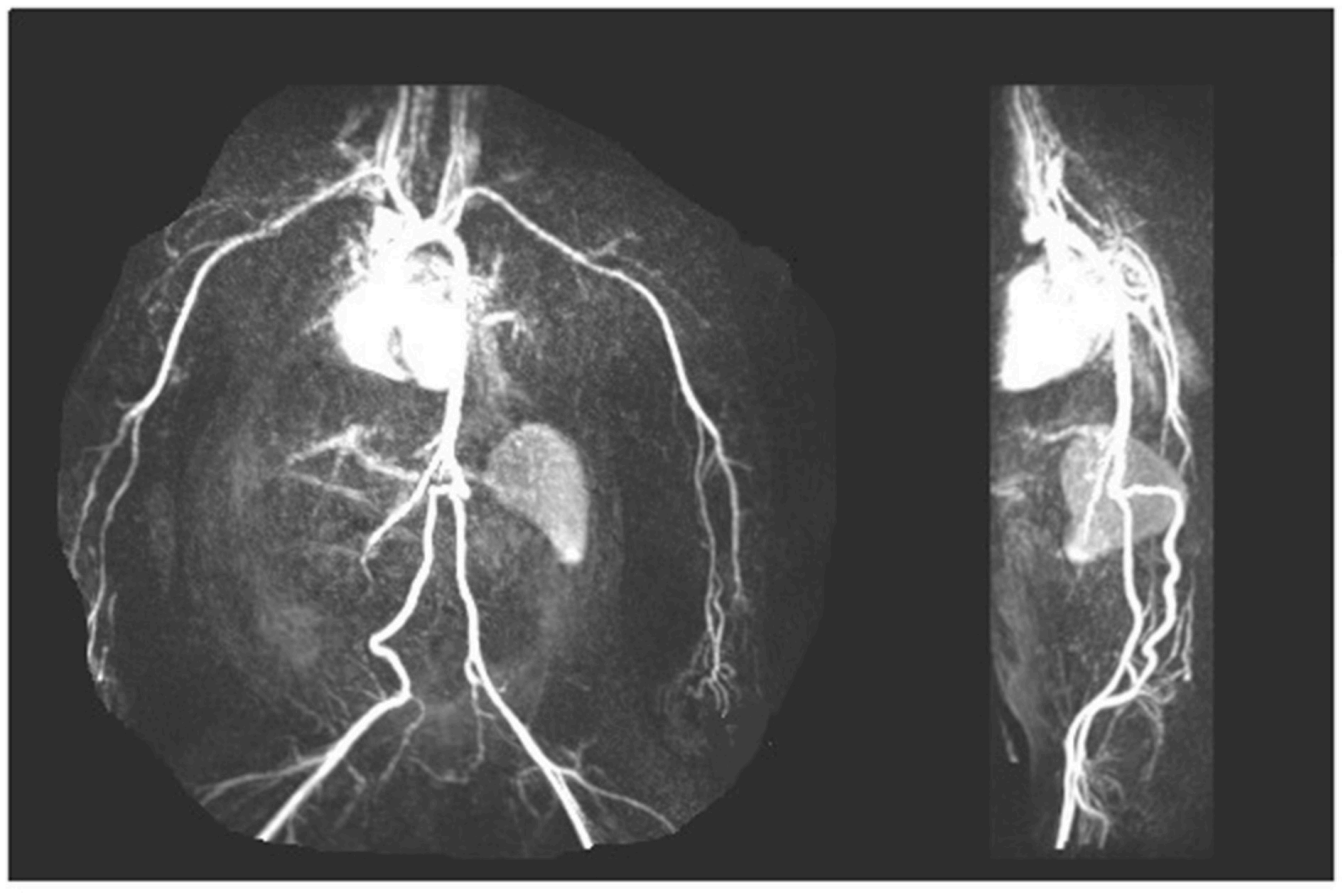
Arteriogram showing early bifurcation of the aorta below the renal vessels with absence of the aorta below the superior mesenteric artery in a CAKUT patient with urogenital sinus anomalies.

Fifteen of 37 males (40%) had fetal surgery for obstructive uropathy and subsequently required urologic surgery to establish and maintain urine flow. Recurrent urinary tract infections was a common complication of persistent urinary obstruction and/or vesicoureteral reflux. Of the 5 females, 4 had VACTERL with complex urogenital sinus anomalies. All required multiple surgeries in preparation for transplantation. All VACTERL subjects progressed to ESRD with prior dialysis, and all had significant vascular anomalies requiring special imaging, including: CT angiograms and MR angiograms. One patient had absence of the aorta below the renal vessels such that traditional renal transplantation would not be possible (Figure 5).

An uncommon association was the occurrence of 2 malignancies. One female with VACTERL was born with a congenital neuroblastoma of the right adrenal, superior to the single functioning dysplastic kidney. It was surgically removed at 9 months of age with preservation of the residual renal function. She was managed conservatively off dialysis until 13 months of age, and then was placed on chronic peritoneal dialysis. The second malignancy was a hepatoblastoma which occurred in an 18 month old male with PBS who was on peritoneal dialysis since 7 months of age. He presented with peritoneal hemorrhage. He had to undergo multiple rounds of chemotherapy after being transitioned to hemodialysis. He underwent a successful combined liver-kidney transplant at 26 months of age and has been in remission with stable liver and renal function with a current age of 5.5 years.

Metabolic bone disease was characterized by sHPT with elevations in the iPTH, and control of the calcium x phosphorus product, acid-base balance, and 25(OH) Vitamin D levels. Of the 42 subjects, elevation in the iPTH level occurred in 28 of 31 (90%) of those investigated. In those with advanced CKD stages, the range in iPTH varied from 79 to 2,744 pg/ml with some becoming refractory to standard treatment with activated vitamin D analogs (calcitriol and paricalcitol). Of those with advanced CKD, 9 of the 27 developed refractory sHPT and were treated with the calcimimetic, cinacalcet, which has been previously reported elsewhere (17).

### Transplantation

During the study period, 12 patients were transplanted at a median age of 3.3 years [IQR: 2.4, 4.7] and a median weight of 14.9 kilograms [IQR: 13.1, 16.3]. Nine (75%) received a deceased donor transplant with one requiring a combined liver-kidney transplant. Five patients received pre-emptive renal transplants at a median age of 3.0 years [IQR: 2.2, 4.4]. Three of the pre-emptive transplants were from living related donors. All have functioning grafts at a current age of 6.8 ± 2.4 years.

## Discussion

In this single center experience of a large birth cohort with severe CAKUT, we provide neonatal predictors of early progression of CKD as well as management paradigms that can promote longevity and growth. Our findings support an emerging multifaceted approach to these complex patients. Close attention to the early assessment of prognostic biomarkers allows better anticipatory guidance and counseling of family and caregivers. This also allows close coordination of surgical and medical management with goals toward preventing infection, preserving and promoting maximum residual renal function with pre-emptive transplantation when feasible.

This is the first report to utilize neonatal CysC in conjunction with peak and nadir SCr as predictive markers of early progression to advanced stages of CKD. Cystatin C does not cross the placental barrier and is independent of muscle mass. It is a more accurate measure of neonatal renal function than is SCr in both term and preterm infants (11). Cord blood CysC ≥3.0 mg/L has been proposed as a predictive measure of mortality and early progression to ESRD in severe bilateral CAKUT (31, 32). This is consistent with our findings in which the CysC≥3.0 mg/L had the highest sensitivity and specificity with the greatest ROC-AUC for prediction to early ESRD. A nadir creatinine between 0.4 and 0.85 mg/dl has been associated with early progression to CKD in patients with PUV (33) which is also consistent with our findings in this birth cohort in which a nadir creatinine ≥0.6 mg/dl had the highest likelihood ratio of predicting progression to advanced CKD.

An important and previously overlooked prognostic indicator for progression to early ESRD is preterm birth before 36 weeks' GA. Interestingly, a low birth weight <2,500 grams was not a prognostic factor. This emphasizes the important role of intra-uterine nephrogenesis which continues in the human until 36 weeks' gestation and post-natal nephrogenesis is not supported by the extra-uterine environment (34). In the fetus with CAKUT, nephrogenesis is *a priori* altered, probably in the very early weeks of gestation. The greatest complement of nephrons, however, is formed during the last trimester of intra-uterine life, making those final weeks extremely important for achieving a maximum complement of nephrons. Our group has recently reported that the prenatal assessment of renal parenchymal area, as a surrogate of nephron mass, using digital software to subtract the degree of associated hydronephrosis, can also predict the severity of renal dysfunction after birth in patients with LUTO who have undergone fetal intervention (18). Hence, the anticipated impairment in nephron endowment may be an important consideration in planning the delivery of infants with severe CAKUT with a focus on trying to extend the gestation to >36 weeks if at all feasible. An additional caveat is that lung maturation is in parallel with nephrogenesis so that longer gestation may avoid additional respiratory complications of preterm birth (35).

Growth is a key indicator of adequate management of infants and children with CKD. Moreover, those with congenital renal disease are the most vulnerable to adult stunting and lifelong bone deformities, as well as cardiovascular complications with a markedly curtailed life expectancy (5–7). In this young cohort, we focused on management paradigms that aggressively engaged the family in feeding, and treatment regimens to maintain metabolic control and adequate nutritional intake. There was early placement of G-tubes in the majority of infants with advanced CKD. Growth hormone was prescribed and maintained in 87% of subjects with improved growth despite interruptions for treatment of the secondary hyperparathyroidism (17). The single most important positive determinant of growth, however, was the renal function. This was apparent in the normal growth of those subjects with early stage CKD and in those post-transplant, particularly those who received pre-emptive transplants with no prior exposure to dialysis.

Gender is an important issue in the analysis for initial and long-term follow-up of severe CAKUT. As in this and other cohorts worldwide, there was a predominance of males. In our cohort the male to female ratio was 8 to 1 with the majority of males having PUV or PBS. Importantly, there were only 5 females, but their anomalies were extremely complex with VACTERL association and urogenital sinus anomalies. As in those male infants with fetal surgical interventions, the female infants with VACTERL were more likely to require multiple surgeries postnatally and to have multiple co-morbidities. Nevertheless, the females had similar outcomes as their male counterparts with severe CAKUT. An important component of the VACTERL-associated anomalies is the high incidence of vascular anomalies that require careful imaging techniques and multidisciplinary planning before embarking on renal transplantation.

The current cohort is unique in that it is a comprehensive assessment of early and severe CAKUT. It is important to recognize the bimodal natural history of this disease with those patients who escape early progression to CKD often developing advanced CKD during adolescence. This is usually related to the development of bladder dysfunction and the natural progression of incipient CKD (36).

The current experience with this cohort provides a platform for considering ethical issues regarding the indications and potential benefits of fetal surgical intervention in the treatment of infants with severe CAKUT diagnosed prenatally. Those who had fetal surgery showed similar progression and potential longevity as those without. Further prospective follow-up is required, preferably in a multi-center collaborative.

## Data Availability

The datasets generated for this study are available on request to the corresponding author.

## Ethics Statement

The study was approved by the University of Miami Institutional Review Board, and patient and family privacy were assured under conditions of the Health Insurance Portability and Accountability Act.

## Author Contributions

All authors contributed to the conception and design of the study. CK and CA wrote the first draft of the manuscript. CK, MD, and CA participated in the critical analysis of the data and the major revisions of the manuscript. All authors contributed to manuscript revision, read and approved the submitted version, including JCI, MC, TC, DSV, WS and JJC.

### Conflict of Interest Statement

The authors declare that the research was conducted in the absence of any commercial or financial relationships that could be construed as a potential conflict of interest.

## References

[1] Weaver DJ Jr Somers MJG Martz K Mitsnefes MM. Clinical outcomes and survival in pediatric patients initiating chronic dialysis: a report of the NAPRTCS registry. Pediatr Nephrol. (2017) 32:2319–30. 10.1007/s00467-017-3759-4 28762101

[2] Chesnaye C Bonthuis M Schafer F Groothoff JW Verrin E Heaf JG. Demographics of paediatric renal replacement therapy in Europe: a report of the ESPN/ERA-EDTA registry. Pediatr Nephrol. (2014) 29:2403–10. 10.1007/s00467-014-2884-6 25039018

[3] van Stralen KJ Borzych-Duzalka D Hataya H Kennedy SE Jager KJ Verrina E. Survival and clinical outcomes of children starting renal replacement therapy in the neonatal period. Kidney Int. (2014) 86:168–74. 10.1038/ki.2013.561 24499775

[4] Hijazi R Abitbol CL Chandar J Seeherunvong W Freundlich M Zilleruelo G. Twenty-five years of infant dialysis: a single center experience. J Pediatr. (2009) 155:111–7. 10.1016/j.jpeds.2009.01.007 19324367

[5] Rees L. Management of the infant with end-stage renal failure. Nephrol Dial Transplant. (2002) 17:1564–7. 10.1093/ndt/17.9.1564 12198206

[6] Sanderson KR Warady BA. End-stage kidney disease in infancy: an educational review. Pediatr Nephrol. (2018). 10.1007/s00467-018-4151-8. [Epub ahead of print]. 30465082 PMC6529305

[7] Kari JA Gonzalez C Ledermann SE Shaw V Rees L. Outcome and growth of infants with severe chronic renal failure. Kidney Int. (2000) 57:1681–7. 10.1046/j.1523-1755.2000.00013.x 10760104

[8] Sanna-Cherchi S Ravani P Corbani V Parodi S Haupt R Piaggio G. Renal outcome in patients with congenital anomalies of the kidney and urinary tract. Kidney Int. (2009) 76:528–33. 10.1038/ki.2009.220 19536081

[9] Danziger P Berman DR Luckritz K Arbour K Laventhal N. Severe congenital anomalies of the kidney and urinary tract: epidemiology can inform ethical decision-making. J Perinatol. (2016) 36:954–959. 10.1038/jp.2016.107 27467564

[10] Schwartz GJ Schneider MF Maier PS Moxey-Mims M Dharnidharka VR Warady BA. Improved equations estimating GFR in children with chronic kidney disease using an immunonephelometric determination of cystatin C. Kidney Int. (2012) 82:445–53. 10.1038/ki.2012.169 22622496 PMC3433576

[11] Abitbol CL Seeherunvong W Galarza MG Katsoufis C Francoeur D Defreitas M. Neonatal kidney size and function in preterm infants: what is a true estimate of glomerular filtration rate? J Pediatr. (2014) 164:1026–31.e2. 10.1016/j.jpeds.2014.01.044 24607244

[12] Zurowska AM Fischbach M Watson AR Edefonti A Stefanidis CJ. European paediatric dialysis working group. clinical practice recommendations for the care of infants with stage 5 chronic kidney disease (CKD5). Pediatr Nephrol. (2013) 28:1739–48. 10.1007/s00467-012-2300-z 23052647 PMC3722439

[13] Kidney Disease: Improving Global Outcomes (KDIGO) CKD-MBD Update Work Group KDIGO 2017 clinical practice guideline update for the diagnosis, evaluation, prevention, and treatment of chronic kidney disease-mineral and bone disorder (CKD-MBD). Kidney Int Suppl. (2017) 7:1–59. 10.1016/j.kisu.2017.04.001 PMC6340919 30675420

[14] National Kidney Foundation K/DOQI clinical practice guidelines for nutrition in children with CKD: 2008 update. bone mineral and vitamin d requirements and therapy. Am J Kidney Dis. (2009) 53:S61 10.1053/j.ajkd.2008.11.017 19231749

[15] Freundlich M Abitbol CL Oral paricalcitol: expanding therapeutic options fo pediatric chronic kidney disease patients. Pediatr Nephrol. (2017) 32:1103–8. 10.1007/s00467-017-3675-7 28451892

[16] Czaya B Seeherunvong W Singh S Yanucil C Ruiz P Quiroz Y. Cardioprotective effects of paricalcitol alone and in combination with FGF23 receptor inhibition in chronic renal failure: experimental and clinical studies. Am J Hypertens. (2019) 32:34–44. 10.1093/ajh/hpy154 30329020 PMC6284753

[17] Arenas Morales AJ DeFreitas MJ Katsoufis CP Seeherunvong W Chandar J Zilleruelo G. Cinacalcet as rescue therapy for refractory hyperparathyroidism in young children with advanced chronic kidney disease. Pediatr Nephrol. (2019) 34:129–35. 10.1007/s00467-018-4055-7 30203374

[18] Moscardi PRM Katsoufis CP Jahromi M Blachman-Braun R DeFreitas MJ Kozakowski K. Prenatal renal parenchymal area as a predictor of early end-stage renal disease in children with vesicoamniotic shunting for lower urinary tract obstruction. J Pediatr Urol. (2018) 14:320.e1–e6. 10.1016/j.jpurol.2018.07.004 30093259

[19] Cisek LJ. holding water: congenital anomalies of the kidney and urinary tract, CKD, and the ongoing role of excellence in plumbing. Adv Chronic Kidney Dis. (2017) 24:357–63. 10.1053/j.ackd.2017.09.012 29229166

[20] National Center for Health Statistics: *Centers for Disease Control and World Health Organization Growth Charts* Available online at: http://www.cdc.gov/growthcharts (accessed September 9, 2010).

[21] Fenton TR Kim JH. A systematic review and meta-analysis to revise the fenton growth chart for preterm infants. BMC Pediatr. (2013) 13:59. 10.1186/1471-2431-13-59 23601190 PMC3637477

[22] Silverstein DM. Growth and nutrition in pediatric chronic kidney disease. Front Pediatr. (2018) 6:205. 10.3389/fped.2018.00205 30155452 PMC6103270

[23] Nelms CL. Optimizing enteral nutrition for growth in pediatric chronic kidney disease (CKD). Front Pediatr. (2018) 6:214. 10.3389/fped.2018.00214 30116725 PMC6083216

[24] Abitbol CL Zilleruelo G Montane B Strauss J. Growth of uremic infants on forced feeding regimens. Pediatr Nephrol. (1993) 7:173–7. 10.1007/BF00864388 8476713

[25] Santos F Moreno ML Neto A Ariceta G Vara J Alonso A. Improvement in growth after 1 year of growth hormone therapy in well-nourished infants with growth retardation secondary to chronic renal failure: results of a multicenter, controlled, randomized, open clinical trial. Clin J Am Soc Nephrol. (2010) 5:1190–7. 10.2215/CJN.07791109 20522533 PMC2893059

[26] Warady BA Iles JN Ariceta G Dehmel B Hidalgo G Jiang X. A randomized, double-blind, placebo-controlled study to assess the efficacy and safety of cinacalcet in pediatric patients with chronic kidney disease and secondary hyperparathyroidism receiving dialysis. Pediatr Nephrol. (2019) 34:475–86. 10.1007/s00467-018-4116-y 30506144

[27] Goebel J. New nephrological frontiers: opportunities and challenges created by fetal care centers. Adv Pediatr. (2017) 64:73–86. 10.1016/j.yapd.2017.03.013 28688600

[28] Hebert SA Swinford RD Hall DR Au JK Bynon JS. Special considerations in pediatric kidney transplantation. Adv Chronic Kidney Dis. (2017) 24:398–404. 10.1053/j.ackd.2017.09.009 29229171

[29] Surabhi VR Menias CO George V Matta E Kaza RK Hasapes J. MDCT and MR urogram spectrum of congenital anomalies of the kidney and urinary tract diagnosed in adulthood. AJR Am J Roentgenol. (2015) 205:W294–304. 10.2214/AJR.14.12867 26295665

[30] Hiorns MP. Imaging of the urinary tract: the role of CT and MRI. Pediatr Nephrol. (2011) 26:59–68. 10.1007/s00467-010-1645-4 20924611 PMC2991216

[31] Tomotaki S Toyoshima K Shimokaze T Shibasaki J Nagafuchi H. Association between cord blood cystatin C levels and early mortality of neonates with congenital abnormalities of the kidney and urinary tract: a single-center, retrospective cohort study. Pediatr Nephrol. (2017) 32:2089–95. 10.1007/s00467-017-3733-1 28681080

[32] Parvex P Combescure C Rodriguez M Birraux J Girardin E. Evaluation and predictive factors of renal function progression using cystatin C and creatinine in neonates born with CAKUT. Clin Nephrol. (2014) 81:338–44. 10.5414/CN108149 24691013

[33] Coleman R King T Nicoara CD Bader M McCarthy L Chandran H. Combined creatinine velocity and nadir creatinine: a reliable predictor of renal outcome in neonatally diagnosed posterior urethral valves. J Pediatr Urol. (2015) 11:214.e1–3. 10.1016/j.jpurol.2015.04.007 26062970

[34] Rodríguez MM Gómez AH Abitbol CL Chandar JJ Duara S Zilleruelo GE. Histomorphometric analysis of postnatal glomerulogenesis in extremely preterm infants. Pediatr Dev Pathol. (2004) 7:17–25. 10.1007/s10024-003-3029-2 15255031

[35] Abitbol CL DeFreitas MJ Strauss J. Assessment of kidney function in preterm infants: lifelong implications. Pediatr Nephrol. (2016) 31:2213–22. 10.1007/s00467-016-3320-x 26846786

[36] Long CJ Bowen DK. Predicting and modifying risk for development of renal failure in boys with posterior urethral valves. Curr Urol Rep. (2018) 19:55. 10.1007/s11934-018-0801-4 29774481

